# Importance of Lignin Coniferaldehyde Residues for Plant Properties and Sustainable Uses

**DOI:** 10.1002/cssc.202001242

**Published:** 2020-08-06

**Authors:** Masanobu Yamamoto, Leonard Blaschek, Elena Subbotina, Shinya Kajita, Edouard Pesquet

**Affiliations:** ^1^ Graduate School of Bio-Applications and Systems Engineering Tokyo University of Agriculture and Technology Tokyo 184-8588 Japan; ^2^ Arrhenius laboratories Department of Ecology, Environment and Plant Sciences Stockholm University 106 91 Stockholm Sweden; ^3^ Arrhenius laboratories, Department of Organic Chemistry Stockholm University 106 91 Stockholm Sweden

**Keywords:** analytical methods, biomass, coniferaldehyde, mutagenesis, polymers

## Abstract

Increases in coniferaldehyde content, a minor lignin residue, significantly improves the sustainable use of plant biomass for feed, pulping, and biorefinery without affecting plant growth and yields. Herein, different analytical methods are compared and validated to distinguish coniferaldehyde from other lignin residues. It is shown that specific genetic pathways regulate amount, linkage, and position of coniferaldehyde within the lignin polymer for each cell type. This specific cellular regulation offers new possibilities for designing plant lignin for novel and targeted industrial uses.

## Graphical abstract

**It′s all in the genes**: Increases in coniferaldehyde residue contents in lignins greatly improve plant biomass sustainable uses for feed, pulping, biogas production, and other biorefinery processes without affecting plant growth or seed/fruit yield. We show that coniferaldehyde incorporation in lignin is regulated by specific genetic processes in different lignified cell types to control its amount, its linkages as well as its position within the lignin polymer.

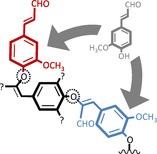

Lignins constitute a class of water‐insoluble phenolic polymers of variable size accumulating in plant cell walls. These polymers have different compositions and concentrations, depending on cell type, their developmental state, and environmental conditions, in order to ensure the chemical and mechanical properties required for the function of each cell type. Lignin is the most abundant renewable source of aromatics representing 15 to 45 % of the dry biomass of plants.^1^, ^2^, ^3^ These large quantities make lignin an ideal resource for future sustainable bio‐economy, but only if we can fully understand, predict and exploit its formation and structure. Lignins are synthesized by the oxidative polymerization of secreted C_6_C_3_ phenylpropenoid compounds differing in their C_6_ aromatic substitution (hydroxyl and methoxyl groups) as well as their C_3_ sidechain terminal function (mostly alcohol) (Scheme 1). Monomeric units with other C_3_ functions are also present in lower amounts, such as acids, esters and aldehydes.^1^, ^2^, ^3^ The main lignin C_6_C_3_ aldehyde monomers derive from coniferaldehyde (C_6_ with 1 hydroxyl and 1 methoxyl group) and sinapaldehyde (C_6_ with 1 hydroxyl and 2 methoxyl groups). It however remains unknown whether *p*‐coumaraldehyde (C_6_ with 1 hydroxyl and C_3_ with aldehyde) also forms residues in developmental lignin. Lignin residues are joined by different linkages, with the β‐*O*‐4, forming an ether linkage between the central C atom of the C_3_ of one residue and the *para O* of the C_6_ of another residue, being the most abundant.^1^, ^2^, ^3^ β‐*O*‐4 linkage with the C_3_ of aldehyde residues results in an unsaturated link, in contrast to C_3_ of alcohol residues (Scheme 1).^2^, ^3^, ^4^ The industrial valorization of lignin aromatic structure, through its depolymerization by methods such as catalytic fractionation, or biogas production,^5^, ^6^ offers promising opportunities to sustainably exploit the lignin in plant biomass by biorefineries. However, the efficiency of these future uses depends on a clear understanding of the different lignin residues incorporated, their distribution and homogeneity between cell types, their position within the polymers as well as their genetic regulation to allow for the optimal utilization of available biomass, and the design of improved plants for biorefineries.

**Scheme 1:**
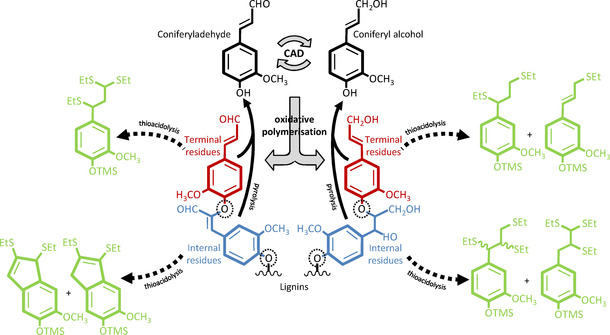
Schematic representation of lignin C_6_C_3_ monomers, coniferaldehyde, and coniferyl alcohol, interconverted by the activity of the NADP^+^/NADPH+H^+^‐dependent CADs as well as β‐*O*‐4‐linked oxidative polymerization lignin products. Enzyme‐catalyzed steps are shown by large grey arrows. Note that terminal residues are indicated in red, internal residues colored in blue, and β‐*O*‐4 linkages indicated by dotted circles. Trimethylsilylated (TMS)‐derivatized thioacidolyzed products corresponding to the different lignin residues are indicated by black dotted lines in green. Black plain arrows indicate pyrolytic products obtained from lignin polymer irrespective of residue position.

Mutagenesis of CINNAMYL ALCOHOL DEHYDROGENASE (CAD) genes has allowed modulating C_6_C_3_ aldehyde residue levels in lignin (Scheme 1). In the herbaceous plant model *Arabidopsis thaliana*, the combined insertional mutagenesis of two CAD paralogs (*cad4*/*c* and *cad5*/*d*) exhibited large increases in lignin aldehyde levels ranging from 35 to 65 % of total measured lignin residues, without altering stem width, biomass weight or fruit yield per plant (Figure 1).^6^, ^7^, ^8^, ^9^ Saccharification and catalytic fractionation yields of *cad4*x*cad5* stem biomass were increased approximately two‐ and threefold respectively, compared to wild‐type (WT) plants.^6^, ^8^ Similar increases of aldehyde residues in lignin by reducing *CAD* expression, using mutagenesis and transgenic approaches in poplar, tobacco, flax, brachypodium, switchgrass, rice and sorghum, have all showed either increased pulping, saccharification and/or biogas yields without affecting plant productivity.^5^, ^10^, ^11^, ^12^, ^13^, ^14^, ^15^, ^16^, ^17^, ^18^ In fact, natural mutants in *CAD*s thrive in the wild and have been readily identified, such as the *CAD*‐null mutant of pine.^19^, ^20^ Natural mutants in *CAD* have also been selected and preferentially used in agriculture more than 100‐years ago, like the Sekizaisou variety of mulberry trees, which improved both silkworm growth and silk quality when used for feed.^21^ The far reaching effects of aldehyde concentration on biomass properties suggest that these residues, despite being considered minor lignin constituents, have a determining role in diversifying the biological functions and industrial uses of lignin in plants. However, although different methods have been previously used to quantify coniferaldehyde residues in lignin, their position, amount and linkage have never been compared to obtain a full picture of how these less abundant residues are accumulated.

**Figure 1:**
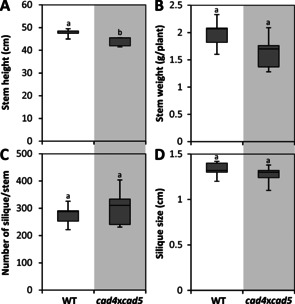
Impact of aldehyde residue over‐accumulation in lignin on *Arabidopsis* plant productivity. Phenotypic differences between wild‐type (WT) and *cad4*x*cad5* double mutant Columbia‐0 plants on stem height (A, *n*=10 plants), stem weight (B, *n*=5 plants), number of fruit per plant (C, *n*=5 plants) as well as fruit size (D, *n*=5 stems and 5 fruits each). Different letters indicate significant differences according to a student *t*‐test with Tukey test (*α*=0.05).

Indeed, synthetic lignins, or dehydrogenation polymers (DHPs), synthesized by directly incubating coniferaldehyde (G_CHO_) monomers with peroxidases (Figure S1 in the Supporting Information), were more hydrophobic and less soluble in a range of solvents than those made from coniferyl alcohol (G_CHOH_).^4^ The artificial lignification of isolated primary cell walls only with G_CHO_ moreover decreased the cross‐bridging between lignin and other cell wall polysaccharides.^22^ Increased lignin aldehyde levels in *Arabidopsis*, poplar and flax stems also decreased their flexural stiffness.^17^, ^23^, ^24^, ^25^ The impact on whole plant physical properties suggests that lignin composition, such as in G_CHO_ residues, alters the overall cell wall organization and its interconnections. Incorporated G_CHO_, but not sinapaldehyde, within the lignin polymer can moreover specifically cross‐react in acid conditions and covalently bind other free phenolic compounds, such as phloroglucinol.^7^ These G_CHO_ residues can also react with NaHSO_3_/Na_2_SO_3_ to form sulfonic acid derivatives.^26^ This suggests that the lateral functionalization of lignin polymers, using internal G_CHO_ residues as anchors, could be used similarly to the lateral functionalization of cellulose using 2,2,6,6‐tetramethylpiperidine‐1‐oxyl (TEMPO) oxidative treatment.^27^ To this end, the reliable quantification of G_CHO_ residue amounts in lignin as well as the quantification of their proportion at the end and/or within the lignin polymer are necessary, but has not yet been demonstrated.

Nuclear magnetic resonance (NMR) analyses of lignin showed that G_CHO_ content in lignin represented ∼7 % in pine, ∼4 % in spruce, ∼8 % in alfalfa and ∼6 % in rice, and reached ∼15 % in *CAD*‐null pine, ∼19 % and ∼88 % respectively in the *cad* mutants of rice and alfalfa.^16^, ^18^, ^19^, ^20^, ^28^ Pyrolysis coupled to gas chromatography and mass spectroscopy (Py−GC/MS), which enables the quantitative measurement of G_CHO_, G_CHOH_ and sinapyl alcohol (S_CHOH_) residues,^29^, ^30^, ^31^, ^32^ showed that total G_CHO_ content in lignin represented ∼9 % in spruce, ∼5 % in *Arabidopsis*, ∼2 % in eucalyptus and ∼0.2 % in poplar.^7^, ^33^, ^34^ The content variability in lignin of G_CHO_, G_CHOH_ and S_CHOH_ residues was further examined using pyrolysis/GC−MS on a set of *Arabidopsis thaliana* mutants affected in one or several genes encoding for enzymes responsible of changing the C_6_ and/or C_3_ parts of lignin monomers (Figure S1). The use of *Arabidopsis* represents an ideal model to quickly investigate multiple genetic engineering strategies and validate analytical methodologies, both transposable to agronomically relevant lignocellulosic feedstock species.^6^, ^7^, ^8^, ^9^, ^10^, ^11^, ^12^, ^13^, ^14^, ^15^, ^16^, ^17^, ^18^, ^19^, ^20^, ^21^ In contrast to total G_CHOH_ and S_CHOH_ residue levels which could only be reduced or annulled compared to WT plants, total G_CHO_ residue content varied by roughly threefold changes in either direction in *Arabidopsis* with specific genetic changes, namely increasing in the *cad4*x*cad5* mutant and decreasing in the *4cl1*x*4cl2* mutant (Figure 2A). *Arabidopsis* natural ecotype variant Wassilewskija (WS) presented ∼60 % more G_CHO_ residues than the Columbia‐0 (Col‐0) ecotype, although their G_CHOH_ and S_CHOH_ residue amounts did not differ (Figure S2). The G_CHO_ over‐accumulation due to the *cad4*x*cad5* mutations was even more accentuated in the WS than in the Col‐0 ecotype, also without affecting G_CHOH_ and S_CHOH_ residue amounts (Figure S2). Overall, our results highlight that the accumulation of G_CHO_ residues in lignin follows a specific regulation differing from the one controlling the abundant G_CHOH_ and S_CHOH_ residue amounts.

**Figure 2:**
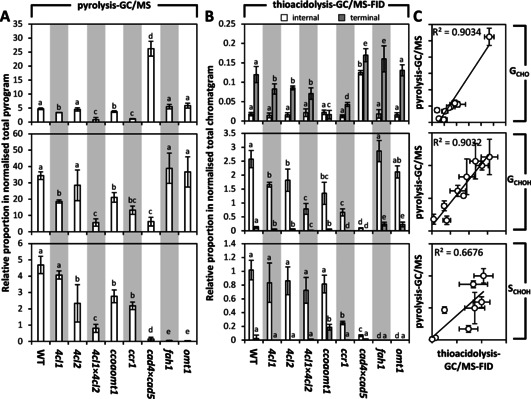
Residue proportions in a set of *Arabidopsis* mutants differently altered in lignin monomer biosynthesis. Analysis of the relative proportion of G_CHO_, G_CHOH_, and S_CHOH_ in lignins of stems using pyrolysis‐GC/MS (A) and thioacidolysis‐GC/MS‐FID (B), with *n*=2–6 independent biological replicates per genotype. Terminal and internal residues correspond to the sum of the different relative peak contributions as shown in Figure 3. The respective position of each mutation in the metabolic pathway is indicated in Figure S1. Different letters for each residue category indicate significant differences according to a one‐way analysis of variance (ANOVA) with Tukey test (with a 95% confidence level *α*=0.05). Linear correlations between the methods for each residue are presented in (C). Note that *R* ^2^ value of the linear regression between thioacidolysis and pyrolysis for G_CHO_ residues is reduced to 0.5685 when removing the *cad4*x*cad5* mutant (extreme value).

We then evaluated the relative positions of G_CHO_ residues in the lignin polymer linked by β‐*O*‐4 (Scheme 1) using thioacidolysis coupled to gas chromatography and detection with mass spectroscopy and flame ionization (thioacidoylsis/GC‐MS‐FID). Lower proportion of β‐*O*‐4 have been reported for DHPs made of only G_CHO_ compared to G_CHOH_.^4^, ^35^, ^36^, ^37^ However, the formation of this link in DHPs also depends on the relative proportion of enzyme to substrate.^38^ We simplified lignin as a linear polymer^39^ with terminal residues at one end keeping their C_3_ sidechain unaltered (Scheme 1). Thioacidolyzed products of the internal and terminal residues were identified using DHPs made of only G_CHOH_, S_CHOH_ or G_CHO_ residues (Figure 3). Terminal and internal residues formed different derivatives depending on their C_6_ and C_3_, allowing the precise distinction between the position of the different monomeric constituents: terminal G_CHO_ as well as internal G_CHOH_ and S_CHOH_ formed different trithioketal derivatives whereas internal G_CHO_ residues formed indene derivatives, and terminal G_CHOH_ and S_CHOH_ formed mono/dithioketal derivatives, as previously reported (Scheme 1 and Figures 3 and S3).^40^, ^41^, ^42^, ^43^, ^44^, ^45^ We then measured the positional proportion of β‐*O*‐4 linked G_CHOH_, S_CHOH_ and G_CHO_ residues in stems of our *Arabidopsis* mutant series with modified lignins. Each type of β‐*O*‐4 linked residues showed specific genetic control: (i) G_CHOH_ content decreased in all mutants except for *fah1* and *omt1*; (ii) S_CHOH_ amounts decreased in *ccr1‐3* and *cad4*x*cad5*, and were absent in *fah1* and *omt1*; and (iii) G_CHO_ levels decreased in *4cl1*, *4cl1*x*4cl2*, *ccoaomt1*, and *ccr1‐3* but increased in *cad4*x*cad5* (Figure 2B). Comparing the levels of β‐*O*‐4 linked G_CHOH_, S_CHOH_ and G_CHO_ determined by thioacidolysis with the total amounts of these residues measured by Py−GC/MS showed different correlation strengths for each residue (Figure 2C). G_CHO_ and G_CHOH_ content correlated strongly between the two methods, suggesting that β‐*O*‐4 represented the main linkage for G_CHO_ and G_CHOH_ in lignin (Figure 2C). In contrast, S_CHOH_ residue content correlated to a lesser extent between the methods, suggesting fewer β‐*O*‐4 linkages exist for S_CHOH_ (Figure 2C). This lower proportion of β‐*O*‐4 for S_CHOH_ residues confirmed previous studies showing higher capacity of S_CHOH_ residues to form other bonds, such as β‐β, in DHPs as well as the low correlation between β‐*O*‐4 proportion and the relative S residue content in poplar natural variants.^46^, ^47^ Altogether, our results showed that different residues are subjected to a specific proportion of β‐*O*‐4 linkages within the lignin polymer. Positional analyses of G_CHOH_, S_CHOH_ and G_CHO_ residues moreover revealed a clear decoupling between the proportion of terminal and internal residues depending on the mutation. WT plants had G_CHO_ terminal residues representing ∼83 % of the normalized chromatogram compared to ∼17 % for internal G_CHO_ residues (Figure 2B). Specific mutants exhibited distinct changes differently affecting the positional proportion: (i) terminal G_CHO_ were specifically decreased by the *4cl1*, *4cl2*, *4cl1*x*4cl2*, *ccoaomt1*, and *ccr1‐3* mutations, but increased in the *fah1* and *cad4*x*cad5* mutants; whereas (ii) internal G_CHO_ were only increased by the *cad4*x*cad5* mutation (Figure 2B). Analyses of the positional proportion of β‐*O*‐4 linked G_CHOH_ and S_CHOH_ residues revealed a different genetic control: (i) terminal G_CHOH_ residues were increased by the *fah1* and *omt1* mutations and decreased in the other mutants, in contrast to internal G_CHOH_ residues which were unaltered in the *fah1* and *omt1* mutations but decreased in the other mutants; and (ii) S_CHOH_ residues were completely absent from the *fah1* and *omt1* mutants, but terminal S_CHOH_ residues increased in the *ccoaomt1* mutant, although internal S_CHOH_ residues only decreased in the *ccr1‐3* and *cad4*x*cad5* mutants (Figure 2B). Overall, our results show that the different lignin monomers are subjected to specific incorporation genetically controlling their amount, their position as well as their linkage‐types within the lignin polymer.

**Figure 3:**
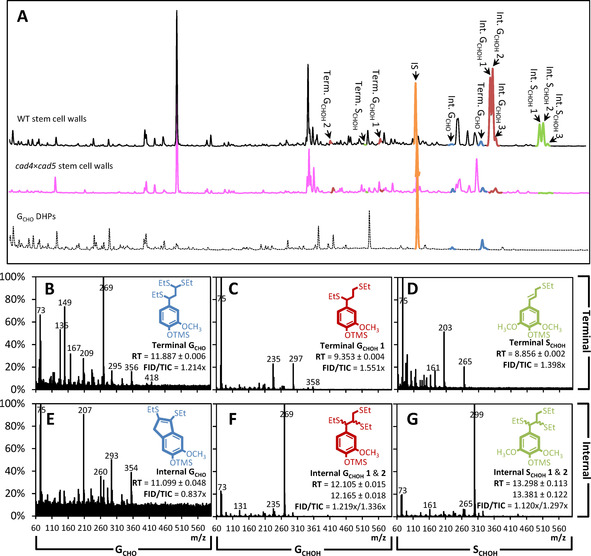
Diagnostic thioacidolyzed compounds deriving from terminal and internal residues of G_CHO_, G_CHOH_, and S_CHOH_ in cell walls of stem tissues. Thioacidolysis chromatogram profiles for WT and *cad4*x*cad5 Arabidopsis* stems compared to G_CHO_ DHPs, in orange the internal standard (IS, tetracosane), in blue G_CHO_ residues, in red G_CHOH_ residues, and in green S_CHOH_ residues (A). Characteristics of diagnostic compounds are presented for terminal (B–D) and internal (E–G) residues of G_CHO_ (B,E), G_CHOH_ (C,F) and S_CHOH_ (D,G) by their *m*/*z*,^40^, ^41^, ^42^, ^43^, ^44^, ^45^ retention time (RT in min) and FID/TIC fold‐ratio (see also Figure S3). Note that internal G_CHOH_ (F) and S_CHOH_ (G) each form two diastereoisomers with different RT (respectively erythro and then threo form in order of elution).^40^, ^41^, ^42^, ^43^, ^44^, ^45^

One essential characteristic of lignin generally overlooked in the context of biomass optimization is its heterogeneity at the cellular and sub‐cellular levels.^1^, ^7^, ^48^ By neglecting this crucial aspect, opportunities are missed to design plant biomass with homogeneous lignin composition to improve its valorization potential. To characterize this cellular heterogeneity of lignin composition, the cell type specific accumulation of G_CHO_, G_CHOH_ and S residues were measured using two in situ quantitative methods. These included the histochemical Wiesner test^7^ as well as Raman confocal microspectroscopy,^49^, ^50^, ^51^ both recently reported to enable quantitative measurement of G_CHO_, G_CHOH_ and S residues in the cell walls of the different cell types (Figure 4A–C). A recent study however showed that the 1625 and 1141 cm^−1^ Raman bands, previously suggested to reflect lignin G_CHO_ residues,^49^, ^50^, ^51^, ^52^, ^53^ did not correlate strongly with either the Wiesner test^7^ or pyrolysis/GC‐MS^51^ quantification of total G_CHO_ residues. Raman microspectra of G_CHO_ residues as monomers and DHPs confirmed the presence of these two characteristic 1625 and 1141 cm^−1^ Raman bands (Figure 4C). Comparison of Raman microspectra obtained from cross‐sections also showed an increased 1625 cm^−1^ and to a lesser extent 1141 cm^−1^ Raman bands in *cad4*x*cad5* mutant compared to WT plants (Figure 4C). We therefore hypothesized that the differences between the Wiesner test and 1625/1141 cm^−1^ Raman bands depended on the position of G_CHO_ residues within the lignin polymer, as they exhibited distinct cell type values (Figure 4D,E). The Wiesner test intensity showed strong correlation with the total β‐*O*‐4 linked G_CHO_ residues measured by thioacidolysis/GC‐MS‐FID, but weaker correlations with terminal or internal G_CHO_ residues (Figure 5). These results confirmed that the Wiesner test detects all G_CHO_ residues in the lignin polymer, thus providing the most precise in situ quantitative method currently available. In contrast, correlation of the 1625 cm^−1^ Raman band did not show any strong association with the total β‐*O*‐4 linked G_CHO_ measured by thioacidolysis/GC‐MS‐FID (Figure 5). Instead, the 1625 cm^−1^ Raman band reflected more the concentration of terminal β‐*O*‐4 linked G_CHO_ residues (Figure 5). This result confirmed previous hypotheses which suggested that the 1625 cm^−1^ band originated predominantly from G_CHO_ units with an unsaturated and unlinked C_3_, such as lignin terminal residues.^53^ The influence of residue position in the lignin polymer on Raman scattering was however specific to G_CHO_ and was not observed for G_CHOH_ or S residues (Figure 5). The 1141 cm^−1^ Raman band had also been used to quantify G_CHO_ residues in milled wood lignin,^52^ but showed weaker correlations than the 1625 cm^−1^ band, probably due to the presence in cross‐sections of other cell wall polymers removed by the milling process (Figure S4). Altogether, our results show that the different in situ imaging methods allowed distinguishing and quantifying G_CHO_ residues in different positions within the lignin polymer at the cellular level.

**Figure 4:**
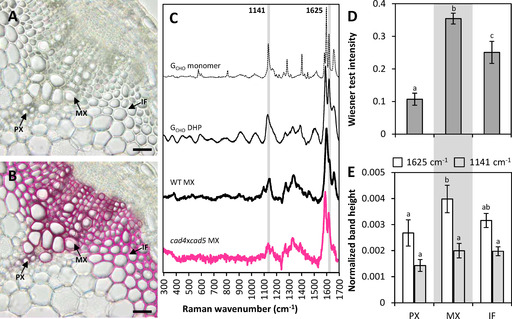
In situ quantitative detection of G_CHO_ content in cell walls of specific cell types in stem cross‐sections of *Arabidopsis*. Sample response before (A) and after (B) staining with to the Wiesner test (phloroglucinol/HCl). Bars=30 μm. Protoxylem vessels (PXs), metaxylem vessels (MXs) and interfascicular fibers (IFs) are indicated by arrows. Standard average Raman spectra of G_CHO_ monomers, DHPs and MXs in WT and *cad4*x*cad5 Arabidopsis* stem cross‐sections (C). The 1141 and 1625 cm^−1^ bands, previously suggested to reflect G_CHO_ residues, are indicated by grey line. Cell type‐specific responses in WT plant cross‐sections for the Wiesner test (D) and Raman (E), *n*=average of each cell type in 3–5 independent biological replicates. Different letters for each category indicate significant differences according to a one‐way ANOVA with Tukey test (*α*=0.05).

**Figure 5:**
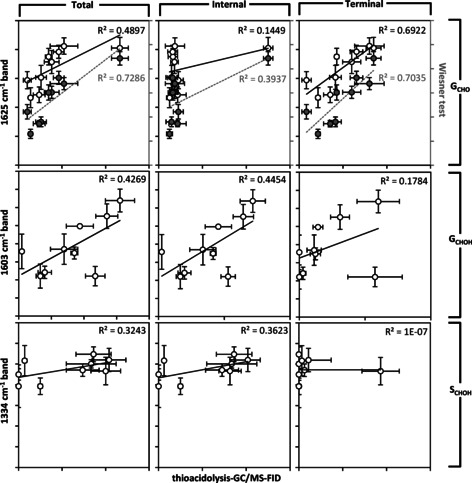
Linear regression analyses between specific Raman band heights and thioacidolysis‐GC/MS‐FID for G_CHO_, G_CHOH_, and S_CHOH_ residues connected by β‐*O*‐4 linkages at different positions with the lignin polymers of stem tissues in a set of *Arabidopsis* with differently modified lignins. Note that instead of Raman, regression analyses between thioacidolysis with Wiesner intensity for G_CHO_ residues are indicated in the right *y* axis of the upper row in grey. Note that the *R* ^2^ value of linear regressions between internal G_CHO_ and 1625 cm^−1^ Raman band is reduced to 0.0001 when removing the *cad4*x*cad5* mutant (extreme value), and between terminal S_CHOH_ and 1334 cm^−1^ Raman band is reduced to 0.1339 when removing the *ccoaomt1* mutant (extreme value).

A recent study has shown that specific genetic regulation controls G_CHO_ residue amounts in the different cell types of *Arabidopsis* and poplar stems.^7^ The accumulation of terminal and total G_CHO_ residues in specific cell types was thus monitored in *Arabidopsis* stems for protoxylem vessels (PX), metaxylem vessels (MX) and interfascicular fibers (IFs). The relative positional proportion of G_CHO_ residues was estimated using the ratio of the 1625 cm^−1^ Raman band, reflecting β‐*O*‐4 linked terminal residues, to the Wiesner test intensity, to account for all G_CHO_ residues. *Arabidopsis* WT plants showed that the three cell types presented different positional proportions in their lignin: PX presented low amount of terminal residue whereas MXs and IFs had similar higher amounts (Figure 6). Analyses of cross‐sections from the *Arabidopsis* mutant series revealed that the genetic regulation controlling the position of G_CHO_ residues differed between the three cell types. PXs and MXs, which have respectively the lowest and highest concentrations of total and terminal G_CHO_ residues in their cell wall (Figure 4D,E),^7^ were not affected by the different genetic regulation altering G_CHO_ biosynthesis (Figure 6). In contrast, IFs were the most susceptible to large changes in the positional proportion of G_CHO_ residues, with large increases in the *4cl1*x*4cl2* and *ccr1‐3* mutants, compared to slight to no decreases in the *cad4*x*cad5*, *fah1* and *omt1* mutants (Figure 6). These results represent an unsuspected discovery on the genetic regulation of the distribution of G_CHO_ residues within the lignin polymer in specific cell types. This specific regulation of G_CHO_ residues in lignin was anticipated from previous analyses using NMR spectroscopy, which only detected β‐*O*‐4 linked G_CHO_ with syringyl (S) residue, but not other guaiacyl (G) residues in CAD down‐regulated angiosperm tobacco, poplar and mulberry – all species with wood composed of more IFs than PXs/MXs.^27^, ^31^, ^54^, ^55^ In contrast, CAD down‐regulated plants from gymnosperms, such as the CAD‐null pine, having wood composed of mostly PXs/MXs, or angiosperms devoid of S residues, such as the *fah1* mutant, are nevertheless capable of linking G_CHO_ residues by β‐*O*‐4 links to other G residues.^8^, ^37^ The proportions of the different lignified cell types vary between plant organs and their developmental state, thus allowing one to harvest biomass with distinct coniferaldehyde profiles. This aspect highlights the advantages of plant biomass as a multipurpose renewable resource for sustainable uses. Although the exact molecular mechanisms enabling the positional control of G_CHO_ residues yet remain unclear, such specific genetic control suggests that the molecular nature by which G_CHO_ monomers are secreted and/or oxidatively polymerized, depending on their positions in the polymer, are differently regulated in each cell type.

**Figure 6:**
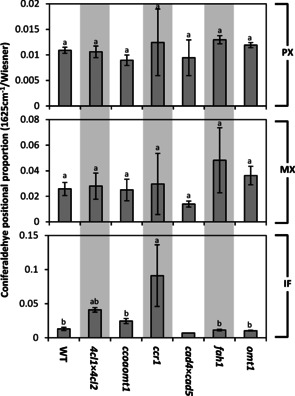
Genetic regulation of the G_CHO_ positional proportion in lignin of different cell types in stem cross‐sections of a set of *Arabidopsis* mutants differently altered in lignin. Cell types include protoxylem vessels (PX), metaxylem vessels (MX), and interfascicular fibers (IFs). Different letters for each residue category indicate significant differences according to a one‐way ANOVA with Tukey test (*α*=0.05), *n*=2–6 cells from 2–3 individual plants per genotype for Raman divided by *n*=cellular average of 5 individual plants per genotype for the Wiesner test.

The extent of the possibilities for the sustainable uses of plant cell wall biomass depends on the compositional homogeneity and predictability of the lignin polymer structure in the feedstock used. Our study details how the amount of G_CHO_ residues in lignin differs between the cell types making up the plant biomass. Such cellular specificity, with large differences in G and S residue levels, had already been reported between MXs and IFs.^1^ These specificities appear to depend on the cell type itself as genetic engineering or monomer feeding to force both angiosperm and gymnosperm MXs to incorporate S residues only slightly changed their lignin composition.^48^ We also showed that the positional distribution of the G_CHO_ residues within the lignin polymer varied between the cell types. It yet remains unknown whether similar cell‐specific regulation mechanisms also exist for the more abundant C_6_C_3_ alcohol monomers. The apparent complexity and evolutionary conservation^48^ of these regulatory systems is understandable from a biological perspective as both the proportions and positions of specific residues will diversify the lignin polymer‘s chemical and mechanical properties to vary its physiological functions. Future studies to decipher the underlying genetic and molecular mechanisms will thus allow defining to which extent plants can be selected or genetically designed to control the G_CHO_ residue distribution within lignin and/or between cell types without hindering agronomical yields for future sustainable uses in biorefineries.

## Experimental Section

**Plant material**: *Arabidopsis thaliana* plants were grown from seeds for 8 weeks on 1 : 3 (v/v) vermiculite/soil in controlled growth chambers under a 16/8 h and 22 °C/18 °C photoperiod with 60 % humidity and 150 μmol m^−2^ s^−1^ illumination using Aura T5 Eco Saver Long Life HO light tubes (AuraLight, Sweden). *Arabidopsis* mutants in the Columbia‐0 background obtained from the European Stock center included: *4cl1‐1* (SALK_14252618), *4cl2‐4* (SALK_11019719), *ccr1‐3* (SALK_123‐68920), *cad4* (SAIL_1265_A0621), *cad5* (SAIL_776_B0621), *ccoaomt1* (SALK_15150722), *fah1‐2* (EMS mutant), *omt1* (SALK_13529024) and double mutants *4cl1‐1*x*4cl2‐4*, and *cad4*x*cad5*. Wassilewskija (WS) wild‐type and *cad4*x*cad5* were provided by Dr. Richard Sibout (INRA Versailles). Basal 4–5 cm of stems were harvested in 70 % ethanol and sectioned, and the rest of the stems were flash‐frozen in liquid nitrogen and stored at ‐20 °C. Transverse cross‐sections were stored in 70 % ethanol to remove protoplasts and extractives and washed twice in ultrapure water prior to imaging.

**Chemicals**: Phenolic compounds used included coniferaldehyde (Aldrich, 382051), coniferyl alcohol (Aldrich, 223735), and sinapyl alcohol (Aldrich, 404586).

**Dehydrogenation polymers (DHPs)**: DHPs were synthesized according to the Zutropf method as previously described.^51^ 10 mL of a solution with 1 mg of horseradish peroxidase (Sigma, P8375‐10KU) in 0.1 M NaHPO_4_ buffer at pH 6 was mixed under magnetic stirring with 10 mL solutions of 14 mM H_2_O_2_ (Sigma‐Aldrich, 95299) and 12 mM of monomer in 3 : 7 methanol/0.1 M NaHPO_4_ buffer at pH 6 at a rate of 0.5 mL h^−1^ using a Legato 200 syringe pump (KdScientific, USA). After 24 h, DHPs in the mixture were isolated by centrifugation at 10000 g for 10 min, the supernatant was removed, and the pellet was washed three times in ultrapure water and freeze‐dried.

**Cell wall isolation**: Extract‐free cell wall material were isolated from stems ground in liquid nitrogen using ceramic mortar and pestle. Proteins and membranes were removed by three washes using vortex mixer agitation with a solution containing 140 mM Tris‐base (Sigma‐Aldrich, T1503), 105 mM tris acetate (Sigma‐Aldrich, T1258), 0.5 mM ethylenediamine tetraacetic acid (EDTA, Scharlau Chemie, AC0965), and 8 % w/v lithium dodecyl sulfate (LDS, Sigma‐Aldrich, L4632) combined with centrifugation (10000 g, 10 min) and the removal of supernatant. Pellets were then successively washed/centrifuged with water, 100 % methanol and finally chloroform/methanol (1 : 1). Pellets were then washed in acetone and air dried overnight.

**Thioacidolysis‐GC/MS‐FID**: Thioacidolysis of plant samples was performed according to Ref. [56]. 5–10 mg of extract‐free cell wall or DHPs and 100 μg internal standard tetracosan (Fuji Film Wako Pure Chem. Ind., 209‐04351) as an internal standard were mixed with a freshly made thioacidolysis reagent containing 87.5 % dioxane (Fuji Film Wako Pure Chem. Ind., 042‐03766), 10 % ethanethiol (97 %, Alfa Aesar, 22585), and 2.5 % boron trifluoride diethyl etherate (>46.5 % BF_3_, Sigma‐Aldrich, 216607) were mixed in a 1 mL screw‐cap reaction vial. The vial cap was screwed on tightly and kept on a sand bath at 100 °C for 4 h with gentle shaking. After cooling the vial in ice water for 5 min, 200 μL of product mixture solution was transferred into a new vial, and 100 μL of 1 M sodium hydrogen carbonate was added. Next, 130 μL of 1 M hydrochloric acid solution was used to adjust the pH to below 3. The resultant solution was extracted three times with 250 μL diethyl ether (Fuji Film Wako Pure Chem. Ind., 055‐01155). The combined organic phase was washed with saturated sodium chloride and then evaporated after drying over anhydrous sodium sulfate. 50 μL of *N*,*O*‐bis(trimethylsilyl)trifluoroacetamide (Sigma‐Aldrich, 15222) and 50 μL anhydrous pyridine (Sigma‐Aldrich, 270970) were added to the vial and kept at 60 °C for 1 h. The mixture was diluted with dichloromethane prior to GC/MS‐FID analysis. GC were performed on a Shimadzu GC‐2010 Plus equipped with a HP‐5 MS capillary column (30 m×0.25 mm×0.25 μm), a FID detector and a Shimadzu GC‐MS‐QP2020 (Shimadzu, Japan). GC/MS‐FID analysis: injector was operated at 250 °C. The column temperature program: 60 °C (2 min), from 60 °C to 260 °C (15 °C ⋅ min^−1^), 260 °C (18 min), from 260 to 300 °C (5 °C ⋅ min^−1^), 300 °C (10 min). Mobile phase used helium at a rate of 1.46 mL min^−1^. The identification of thioacidolyzed derivatives of lignin monomers was based on previous publications.^40^, ^41^, ^42^, ^43^, ^44^, ^45^ Chromatograms were analyzed using Openchrom (https://lablicate.com/platform/openchrom) and proportion of each residue was expressed as their contribution to the total chromatogram area relatively to the initial cell wall weight and internal standard.

**Pyrolysis‐GC/MS**: Pyrolysis GC‐MS analysis was performed according to Ref. [32] on 60 μg (±10 μg) of freeze‐dried ball‐milled stem samples using a pyrolyzer equipped with an autosampler (PY‐2020iD and AS‐1020E, Frontier Lab, Japan) connected to a GC/MS (7890A/5975C; Agilent Technologies AB, Sweden). Pyrolytic peak identification of lignin monomers was based on previous publications.^29^, ^30^, ^31^, ^32^ Proportion of each residue was expressed as their contribution to the total pyrogram area.

**In situ quantitative lignin analysis**: Quantitative Wiesner data was taken from Ref. [7], and is available in the Supporting Information of that publication. Briefly, 50 μm stem cross‐sections were imaged before and after staining with 0.5 % phloroglucinol (Sigma, P3502) in 1 : 1 ethanol/HCl (37 %). The acquired images were transformed into absorbance using ImageJ, aligned, and measured in 50 circular points per plant and cell type. Finally, the unstained background absorbance of each point was subtracted from the stained absorbance. Quantitative Raman microspectroscopy data was partly taken from Ref. [51] and extended using the same experimental setup. Briefly, spectra from stem cross‐sections were acquired using a Raman Touch‐VIS‐NIR (Nanophoton, Japan) equipped with a 532 nm laser. Spectra (1.6 cm^−1^ resolution) were baseline corrected using an asymmetric least‐squares algorithm and normalized to the total Raman signal (area under the curve) between 300 and 1700 cm^−1^.

## Conflict of interest

The authors declare no conflict of interest.

## Supporting information

As a service to our authors and readers, this journal provides supporting information supplied by the authors. Such materials are peer reviewed and may be re‐organized for online delivery, but are not copy‐edited or typeset. Technical support issues arising from supporting information (other than missing files) should be addressed to the authors.

Supplementary
