# Supplementary material for: Importance of Lignin Coniferaldehyde Residues for Plant Properties and Sustainable Uses
Source: ChemSusChem. 2020 Aug 6;13(17):4400–8. doi: 10.1002/cssc.202001242 (PMC7539997; doi:10.1002/cssc.202001242)
Supplement: Supplementary file 1 — Supplementary [file CSSC-13-4400-s001.pdf]

ChemSusChem

Supporting Information

Importance of Lignin Coniferaldehyde Residues for Plant Properties and Sustainable Uses

Masanobu Yamamoto, Leonard Blaschek, Elena Subbotina, Shinya Kajita, and
Edouard Pesquet*© 2020 The Authors. Published by Wiley-VCH GmbH.
This is an open access article under the terms of the Creative Commons Attribution License, which permits use, distribution and reproduction in any medium, provided the original work is properly cited.This publication is part of a Special Issue focusing on "Lignin Valorization: From Theory to Practice". Please visit the issue at .

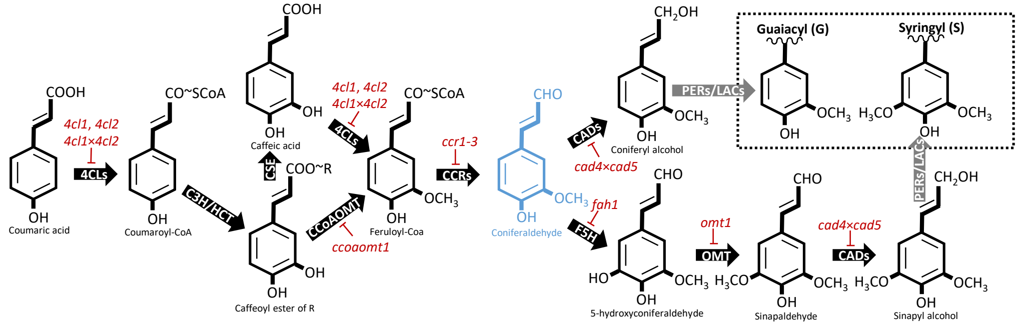

Figure S1. Schematic representation of conventional lignin monomer biosynthesis (black arrows) and polymerization (grey arrows) processes. Note that mutants used in this study are presented in red for 4-COUMARATE:CoA LIGASEs (4CLs), CAFFEOYL-CoA *O*-METHYLTRANSFERASE 1 (CCoAOMT1), CINNAMOYL-COA REDUCTASEs (CCRs), CINNAMYL ALCOHOL DEHYDROGENASEs (CADs), FERULATE-5 HYDROXYLASE (F5H), *O*-METHYLTRANSFERASE (OMT). Other important step in lignin formation include COUMARATE-3-HYDROXYLASE and SHIKIMATE/QUINATE HYDROXYCOUMAROYLTRANSFERASE (C3H/HCT), CAFFEOYL-SHIKIMATE ESTERASE (CSE), PEROXIDASEs (PERs) AND LACCASEs (LACs). Note that coniferaldehyde is indicated in blue.

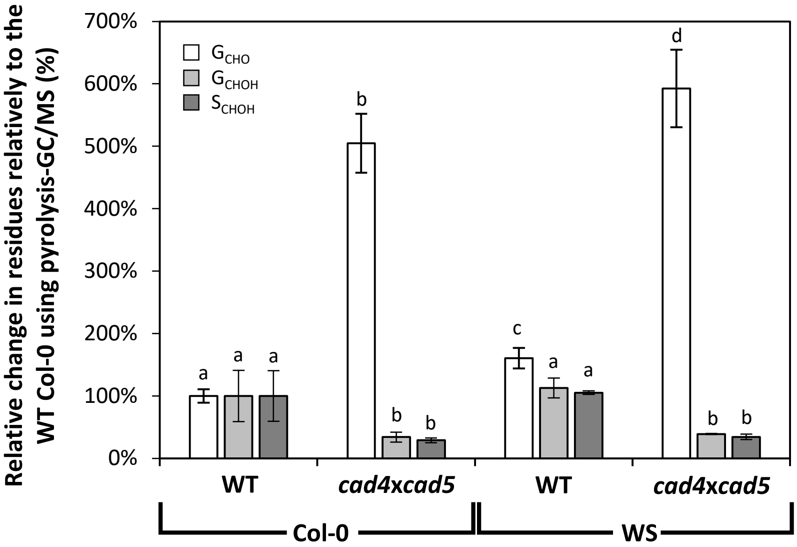

Figure S2. Influence of natural diversity on the proportion of coniferaldehyde (G_{CHO}), coniferyl alcohol (G_{CHOH}) and sinapyl alcohol (S_{CHOH}) in lignin of *Arabidopsis* stem tissues using pyrolysis-GC/MS. Data is expressed as a percentage of wild-type (WT) plants of the Columbia 0 (Col-0) background for each residue compared to Wassilewskija (WS) and/or *cad4*x*cad5* mutations (n = 3 independent biological replicates per genotype). Different letters for each residue category indicate significant differences using one-way ANOVA with Tukey test.

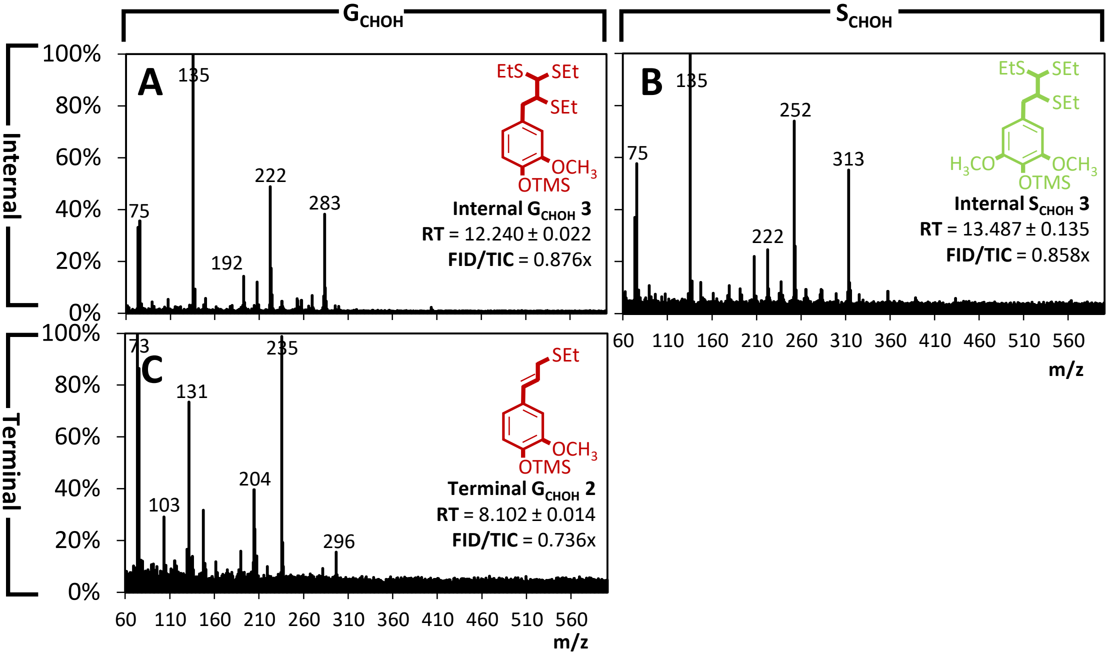

Figure S3. Diagnostic thioacidolysed compounds deriving from terminal and internal residues of coniferyl alcohol (G_{CHOH}) and sinapyl alcohol (S_{CHOH}) in cell walls of stem tissues. Characteristics of diagnostic compound are presented for internal (A-B) and terminal (C) residues of G_{CHOH} (A,C) and S_{CHOH} (B) by their m/z[40-45], retention time (RT in min) and FID/TIC ratio.

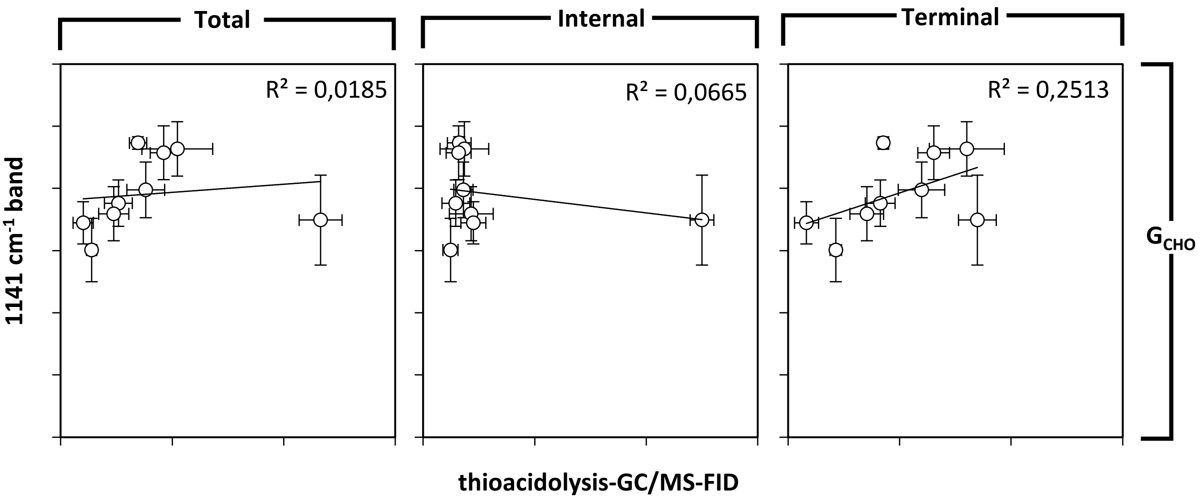

Figure S4. Linear correlation analyses between the 1141 cm^{-1} Raman band height and thioacidolysis-GC/MS-FID for coniferaldehyde (G_{CHO}) residues connected by ether C-O-C linkages at different position with the lignin polymers of stem tissues in a set of *Arabidopsis* with modified lignins.
